# Only Acute but Not Chronic Thrombocytopenia Protects Mice against Left Ventricular Dysfunction after Acute Myocardial Infarction

**DOI:** 10.3390/cells11213500

**Published:** 2022-11-04

**Authors:** Friedrich Reusswig, Amin Polzin, Meike Klier, Matthias Achim Dille, Aysel Ayhan, Marcel Benkhoff, Celina Lersch, Anika Prinz, Simone Gorressen, Jens Walter Fischer, Malte Kelm, Margitta Elvers

**Affiliations:** 1 Heinrich-Heine University Medical Center, Department of Vascular and Endovascular Surgery, Experimental Vascular Medicine, 40225 Düsseldorf, Germany; 2 Heinrich-Heine University Medical Center, Department of Cardiology, Pulmonology and Angiology, 40225 Düsseldorf, Germany; 3 Institute for Pharmacology and Clinical Pharmacology, Heinrich-Heine University, 40225 Düsseldorf, Germany

**Keywords:** platelets, myocardial infarction, inflammation, remodeling, scar formation

## Abstract

Background: Platelets are major players of thrombosis and inflammation after acute myocardial infarction (AMI). The impact of thrombocytopenia on platelet-induced cellular processes post AMI is not well defined. Methods: The left anterior descending artery was ligated in C57/Bl6 mice and in two thrombocytopenic mouse models to induce AMI. Results: Platelets from STEMI patients and from C57/Bl6 mice displayed enhanced platelet activation after AMI. This allows platelets to migrate into the infarct but not into the remote zone of the left ventricle. Acute thrombocytopenia by antibody-induced platelet depletion resulted in reduced infarct size and improved cardiac function 24 h and 21 days post AMI. This was due to reduced platelet-mediated inflammation after 24 h and reduced scar formation after 21 days post AMI. The collagen composition and interstitial collagen content in the left ventricle were altered due to platelet interaction with cardiac fibroblasts. Acute inflammation was also significantly reduced in *Mpl^−/−^* mice with chronic thrombocytopenia, but cardiac remodeling was unaltered. Consequently, left ventricular function, infarct size and scar formation in *Mpl^−/−^* mice were comparable to controls. Conclusion: This study discovers a novel role for platelets in cardiac remodeling and reveals that acute but not chronic thrombocytopenia protects left ventricular function post AMI.

## 1. Introduction

Platelets are small, anucleated blood cells and play an important role in hemostasis after vessel injury, but are also causative for vessel occlusion and ischemic events under pathological conditions, as observed in patients with acute myocardial infarction (AMI) [1]. According to the World Health Organization (WHO) ischemic heart diseases are still the global leading cause of death. According to the electrocardiogram of patients, AMI can be classified into non-STEMI (non-ST-segment elevation myocardial infarction) or STEMI, which differs in the incidence and in short-term case fatality rates for myocardial infarction [2]. The guidelines recommended for STEMI treatment are to re-canalize the obstructed coronary vessel by percutaneous coronary intervention (PCI) [3]. However, recanalization of the vessel can lead to ischemia/reperfusion (I/R) injury, which can cause additional cardiac damage in patients with AMI [4]. Antiplatelet therapy is the backbone of secondary prevention post-AMI, because inhibition of platelet activation, e.g., by P2Y12 inhibitors and aspirin, is crucial to prevent recurrent arterial thrombosis and ischemic events [5,6]. Antiplatelet interventions in mice with experimental AMI leads to the inhibition of inflammatory cell recruitment into the infarcted myocardium [7]. Besides these beneficial effects of antiplatelet interventions, an enhanced risk for bleeding complications in mouse models and in patients was observed [8,9]. In particular, patients with thrombocytopenia suffer from increased bleeding complications and enhanced mortality after AMI [10,11]. Other studies provide evidence for a correlation of idiopathic thrombocytopenic purpura with lower rates of acute ST-elevation myocardial infarction (MI) [12]. Experimental mouse models of AMI with genetic loss or blockage of different platelet surface proteins such as Glycoprotein (GP) VI [13,14], P-selectin [15] or protease-activated receptor (PAR)-4 [16] provide evidence for a dominant role of platelet activation in AMI because decreased infarct size and reduced inflammatory responses were observed in these mice, thus emphasizing the important role of platelets for the pathogenesis of AMI. A role for platelets in coronary thrombosis and the inflammatory response after AMI is known for many years [15,17,18,19]. Platelets modify inflammation by direct interaction with endothelial cells or leukocytes or by the release of their cargo secreted from their granules [17,20,21]. Furthermore, released platelet mediators promote platelet activation and thrombosis by autocrine or paracrine amplification pathways, but can also regulate diverse cellular functions within the microenvironment [20]. Growing evidence in the last years suggests that platelets might influence different processes after AMI, such as inflammation, angiogenesis and cardiac repair, and thus play a bivalent role with protective and damaging characteristics [20,22,23,24]. 

Using an acute and a chronic thrombocytopenic mouse model, we here investigated the consequences of thrombocytopenia on inflammation, cardiac remodeling, scar formation and left ventricular function, thereby emphasizing the crucial role of platelets in these processes. Importantly, acute thrombocytopenia reduced inflammation and improved cardiac remodeling, leading to decreased infarct size and improved cardiac function. while chronic thrombocytopenia did not alter infarct/scar size and left ventricular function after AMI. 

## 2. Methods

### 2.1. Animals

Animal studies were performed in accordance with the guidelines of the European Parliament for the use of living animals in scientific studies and in accordance with the German law for protection of animals. The protocol was approved by Heinrich-Heine-University Animal Care Committee and by the district government of North-Rhine-Westphalia (LANUV; NRW; Permit Number 84-02.04.2015.A558; 81-02.04.2019.A270; 84-02.04.2017.A440). C57BL/6J mice were bought from Janvier (Le Genest-Saint-Isle, France) and thrombocytopenia was induced by treatment with GPIb-antibody or adequate IgG-antibody as control (description see Section 2.2). In addition, mice with genetically induced thrombocytopenia were used (*Mpl^−/−^* mice) [25]. A global double-fluorescent Cre reporter mouse (Gt(ROSA)26Sor^tm4(ACTB-tdTomato,-EGFP)luo^/J) from Charles River (Cologne, Germany), described from Muzumdar et al. [26], was used and bred to PF4-Cre recombinase expressing mouse strain (C57BL/6-Tg(PF4-cre)Q3Rsko/J; The Jackson Laboratory, Bar Harbor, ME, USA), which allows a megakaryocyte and platelet specific gene knockout [27]. The resulting offspring have the mT cassette (red fluorescent) deleted in platelets, allowing expression of the downstream located membrane-targeted eGFP (mG, green fluorescent) cassette. 

### 2.2. Antibody Induced Thrombocytopenia in Mice

For the induction of thrombocytopenic mice, platelet depletion was induced by injection of a GPIb antibody (antibody for mouse platelet depletion, #R300, polyclonal anti-GPIb alpha, Emfret, Eibelstadt, Germany) or the corresponding IgG-control antibody (#C301, polyclonal non-immune rat immunoglobulins (IgG), Emfret, Eibelstadt, Germany) 24 h before the LAD was ligated and at the following days as indicated. According to the data sheet (#R300, Emfret), mice received 2 µg/g or 4 µg/g body weight of the antibody solved in sterile PBS to deplete platelets in these mice. Platelet depletion, general blood cell count and isolated platelet count at different time points after I/R were monitored by using the automated hematology analyzer Sysmex (Sysmex Corporation, Kobe, Japan). 

### 2.3. Experimental Model of Acute Myocardial Infarction (AMI) and Reperfusion in Mice

A closed-chest model of reperfused myocardial infarction was used in order to reduce surgical trauma and consequent inflammatory reaction from the intervention and the antibody injection following I/R [28]. 10 to 12-week-old male mice were anesthetized with Ketamin (100 mg/kg body weight, Ketaset^®^, company: Zoetis, Malakoff, France) and Xylazin (10 mg/kg body weight, Xylazin, company: WDT, Ulft, The Netherlands) by a singular intraperitoneal (i.p.) injection before surgery. Euthanasia was performed by cervical dislocation.

After progressing successfully through anesthesia, the left anterior descending artery (LAD) was ligated for 60 min to induce MI 3 days post instrumentation. Coronary occlusion was achieved by gently pulling the applied suture tight until ST-elevation appeared on the ECG. Reperfusion was confirmed by resolution of ST-elevation. After 24 h of reperfusion, hearts were removed and stained with TTC/Evans Blue–solution to stain the damaged area, separated in the area at risk (ischemic area) and the infarcted area. The ratios of the different areas were quantified digitally by video planimetry. To determine left ventricular function after MI, echocardiography was performed at different time points after I/R using Vevo 2100 ultrasound machine (VisualSonics Inc., Bothell, WA, USA) to measure different parameters, e.g., ejection fraction (%), cardiac output (mL/min), fractional shortening (%) and stroke volume (µL) with corresponding software. In another set of experiments, perfusion was performed for indicated time points (5 days, 21 days). For details, please see Supplementary Figure S1.

### 2.4. Experiments with Human Blood

Experiments with human blood were reviewed and approved by the Ethics Committee of the Heinrich-Heine-University. Subjects provided informed consent prior to their participation in the study (patients’ consent). The experiments conform to the principles outlined in the Declaration of Helsinki.

### 2.5. Isolation of Cardiac Fibroblasts and Incubation with Platelet Supernatant

Primary cardiac fibroblasts were isolated from 10–12-week-old wildtype mice as previously described [29]. In brief, the cells were isolated via retrograde perfusion with collagenase type I (Worthington, OH, USA). After filtering and pelleting the cells were plated in T75 flasks. Non-adherent cells and debris were removed by aspiration after 4 h. Adherent cells were cultured for 48 h in growth media consisting of 5.5 mM glucose DMEM supplemented with 10% FBS, 100 U/mL penicillin and 100 µg/mL streptomycin (Gibco Life Technologies, Carlsbad, CA, USA). After 48 h of incubation, fibroblasts were washed extensively with fresh media to remove debris and non-adherent cells. After further 48 h of incubation, cells were trypsinized and plated on 6-well plates with a density of 12,000 cells per cm^2^. Before, incubation with platelet supernatant cardiac fibroblast were synchronized for 48 h in 1% FBS. 

To produce platelet supernatant, murine platelets were isolated from wildtype animals, as previously described [30]. Isolated platelets were incubated with or without 5 µg/mL CRP (collagen-related peptide) in Tyrode’s buffer for 5 min with subsequent centrifugation to collect supernatant from resting and activated platelets. Cardiac fibroblasts were incubated with supernatant from 100 Mio platelets in a 1:10 dilution with growths medium for 24 h and 72 h. Fibroblast treated with Tyrode’s buffer without platelets was used as control. 

### 2.6. Flow Cytometric Analysis of Blood Cells

To analyze MI-evoked alteration of the expression of different surface parameters of platelets and interacting cells, the expression of different parameters was measured by flow cytometry.

For determining P-Selectin expression or activation of the integrin α_IIb_β_3_ whole blood was acquired by puncturing the retrobulbar vein plexus of anesthetized mice. 2–3% isofluran (Isofluran-Piramal, Piramal Critical Care B.V., Voorschoten, The Netherlands) was used for anesthesia of mice. Blood was washed three times by centrifugation with Tyrode’s buffer. Platelets were stimulated by classical agonists (CRP, Richard Farndale, University of Cambridge, United Kingdom; PAR-4 activating peptide, AYPGKF, JPT Peptide Technologies) and incubated with FITC-labeled antibody for P-Selectin (Wug.E9-FITC, Emfret Analytics, Eibelstadt, Germany) and PE-labeled antibody (JON/A-PE, Emfret Analytics, Eibelstadt, Germany) for activated integrin α_IIb_β_3_. The MFI (mean of fluorescence intensity) was determined to represent the exposition of the indicated platelet surface parameters.

For detection of the PS exposure Cy™5 AnnexinV (BD Biosciences, Heidelberg, Germany) staining was conducted while binding buffer (10 mM Hepes, 140 mM NaCl, 2.5 mM CaCl_2_, pH 7.4) was used instead of PBS and CD42 was used as platelet specific marker.

Either platelet- neutrophil- or platelet- leukocyte aggregate formation was measured before 24 h, and 72 h post AMI via flow cytometry. Heparinized blood was washed twice with Tyrode’s buffer, centrifuged at 650 g for 5 min, supernatant was wasted and only the cell rich pellet was used for analysis. Pellet was incubated with PE-labeled antibody for platelets (GPIb-PE, Emfret, Eibelstadt, Germany) and APC-labeled antibodies for either neutrophils (Ly6G-APC, Biolegend, San Diego, CA, USA) or leucocytes (CD45-APC, BD Bioscience, Franklin Lakes, NJ, USA). The MFI (mean of fluorescence intensity) is representing the fluorescence intensity of the immune cell signaling gated to the immune cell/platelets signal.

To analyze if neutrophils are activated after myocardial infarction, Mac-1 expression was determined. Before and 24 h after MI heparinized blood was washed twice by centrifuging 5 min for 650 g. Supernatant was wasted and the pellet was incubated with Ly-6G Dylight 488 (Leinco, St. Louis, CA, USA) to label leukocytes and APC- labeled CD11b- antibody (MAC-1, Becton Dickinson GmbH, Heidelberg, Germany) for 15 min at room temperature. The MFI was determined and represented MAC-1 exposition of activated neutrophils.

### 2.7. Enzyme-Linked Immunosorbent Assay (ELISA)

For quantification of IL-1β in plasma of mice at different time points after myocardial infarction, heparinized blood was centrifuged 10 min for 650× *g* to retrieve the plasma. The cytokine amount was measured by IL-1β enzyme-linked immunosorbent assay (ELISA; DuoSet Mouse IL-1ß/IL-1F2, R&D systems, Minneapolis, MN, USA) following the manufacturer’s protocol.

Plasma levels from CCS and STEMI patients were analyzed for soluble GPVI (My Biosource, San Diego, CA, USA) via ELISA following the manufacturer´s protocol.

### 2.8. Immunohistochemistry of Cardiac Sections

At different time points after ischemia/reperfusion, hearts were flushed with cold heparin solution (20 U/mL, Roche, Basel, Switzerland), removed, paraffin embedded and cut into 5 µm sections. 

24 h after myocardial infarction, paraffin-embedded heart sections were stained by Hematoxylin/Eosin (HE) solution (Carl Roth, Karlsruhe, Germany) and the total number of cells migrated into the infarcted area was counted per visual field. Data are shown per *10^3^/mm^2^.

To analyze the number of immune cells after myocardial infarction, paraffin-embedded heart section was stained with Mac-3 or Ly6G antibody (Cell Signaling technology, Danvers, MA, USA) as primary antibody, respectively, followed by labeling with streptavidin biotin/horseradish peroxidase (LSAB2 System HRP, Dako, Santa Clara, CA, USA) and Diaminobenzidine (DAB)—Chromogen (Dako, Santa Clara, CA, USA), performed as standard protocol. 

### 2.9. Collagen-Staining of Cardiac Sections

For collagen staining 21 days after myocardial infarction, heart sections were prepared as mentioned before (see Section 2.6). To determine the infarct size, the heart section was stained by Gomori´s one step trichrome staining. The infarct size was expressed as the percentage of the total left ventricular (LV) area. For quantification of the interstitial collagen deposition, cardiac sections were stained by Picrosirius red staining (Morphisto, Frankfurt am Main, Germany) and Celestineblue-solution (Sigma, St. Louis, CA, USA) was used to stain the nuclei. Interstitial collagen was measured in percent by area fraction. Collagen density was analyzed by polarized light microscopy and evaluated by Image J software (Version number V 1.8.0, creator: Wayne Rasband, National Institutes of Health and the Laboratory for Optical and Computational Instrumentation LOCI, University of Wisconsin, (Madison, WI, USA)).

### 2.10. Immunofluorescence (IF)-Staining of Heart Sections

At different time points after ischemia/reperfusion hearts were flushed with cold heparin solution (20 U/mL, Roche, Basel, Switzerland), removed, embedded in OCT compound (Tissue-Tek, Sakura Finetek Europe B.V., Alphen aan den Rijn, The Netherlands) with overnight freezing at −70 °C and cut with cryotome (Leica CM3050 S Cryostat, Leica Microsystems, Wetzlar, Germany) to 5 µm sections. For IF-staining for αSMA-staining the hearts were embedded in paraffin and cut into 5 µm sections. 

To distinguish remote and infarcted area in heart cryosections after myocardial infarction WGA (Wheat Germ Agglutinin, Alexa Fluor™ 647 Conjugate, Thermo Fisher Scientific Inc., Waltham, MA, USA, 1.0 mg/mL)—staining was performed to visualize intact cell membranes as an indicator for remote area and damaged cells as an indicator for the infarcted area. The heart section was fixed with 4 °C acetone, permeabilized by Proteinase K and RNase I (Thermo Fisher Scientific Inc., Waltham, MA, USA) and WGA-staining was performed following the manufacturer’s protocol. DAPI-staining was performed as described before.

To label endothelia cells of vessels within the heart-sections, IF-staining for PECAM-1 (Platelet endothelial cell adhesion molecule-1) was performed. After fixation heart-sections were blocked by solution of 5% goat serum/5% bovine serum albumin in PBS for 30 min and incubated with either PECAM-1 (Purified Rat anti-mouse CD31, BD, Frankling Lakes, NJ, USA, 1:75)-antibody or isotype control (Rat IgG2b kappa, eB149/10H5; Thermo Fisher Scientific Inc., Waltham, MA, USA, 1:50) 1 h at RT. To label primary antibodies incubation with Alexa Fluor 647 goat anti rat-IgG (Life technologies, Waltham, MA, USA, 1:100) for 1 h at RT followed. Additional DAPI-staining was performed as described before. 

To label myofibroblasts, αSMA-staining were conducted in sections of paraffin embedded hearts of IgG injected and platelet depleted mice 21 days post AMI. After fixation heart-sections were blocked by solution of 5% goat serum in PBS for 1 h and incubated with αSMA (Purified Rabbit anti-mouse αSMA, Abcam, Cambridge, UK, 1:100)-antibody or isotype control (Rat IgG2b kappa, eB149/10H5; Thermo Fisher Scientific Inc., Waltham, MA, USA, 1:100) o/n at 4 °C. To label primary antibodies incubation with Alexa Fluor 555 goat anti rabbit-IgG (Life technologies, Waltham, MA, USA, 1:100) for 1 h at RT followed. Additional DAPI-staining was performed as described before.

All fluorescence images were acquired using a confocal microscope (Carl Zeiss, Oberkochen, Germany) and supporting software.

### 2.11. Quantitative Real-Time Polymerase Chain Reaction (qRT-PCR)

RNA from primary cardiac fibroblasts were isolated by Trizol/chloroform extraction and purification by RNAeasy Mini Kit (Qiagen, Hilden, Germany) following the manufacturer´s protocol. Reverse transcription and quantitative PCR were performed as described above. Following oligonucleotide primers were used as marker for activated fibroblasts: *Acta2* for 5′ATGGAGTCAGCGGGCATC′3; rev 5′CGTTCTGGAGGGGCAATGAT′3; *Col1α1* for 5′AGGCGAAGGCAACAGTCG′3; rev 5′TTTACACGAAGCAGGCAGGG′3; *Col3α1* for 5′GCCTCCCAGAACATTACATACC′3; rev 5′CTTGCTCCATTCC-CCAGTGT′3; *Postn* for 5′TTCGTGGCAGCACCTTCAAA′3; rev 5′GTC-ACCGTTTCGCCTTCTTT′3; *Cthrc1* for 5′GCTGTCAGCGCTGGTATTTT′3; rev 5′ACCCAGATGGCCACATCTAC′3. Gene expressions were calculated relative to the housekeeping gene *Rpl32:* for 5′GCCCAAGATCGTCAAAAAGA′3; rev 5′ATT-GTGGACCAGGAACTTGC′3.

### 2.12. Study Population and Light Transmission Aggregometry (LTA)

Patients who were admitted to the University Hospital Düsseldorf were checked for eligibility. Inclusion criteria were ST-elevation (STEMI) or chronic coronary syndrome (CCS) in existing coronary artery disease (CAD). Exclusion criteria were the following: Palliative situation (life expectancy lower than 8 weeks), pregnancy, cognitive impairment, dementia, severe chronic kidney disease (stage 3b-5), severe liver dysfunction, and patients with platelets disorders.

Eligible patients were informed about the content of the study and asked to voluntarily participate. Written consent was obtained from all patients. The study is approved by the Heinrich-Heine University Düsseldorf ethics committee.

Blood sampling was conducted directly at presentation with ST-elevation myocardial infarction in the chest pain unit before percutanous coronary intervention. Patients were pretreated with acetylicsalicylic acid by the emergency doctor before blood sampling. P2Y12 inhibition was initiated before proceeding to percutanous coronary intervention. Therefore, patients were P2Y12 naiive at the timepoint of blood sampling.

Blood was drawn from 30 STEMI and 18 CCS patients into 2.7 mL 0.1 M sodium citrate tubes (BD Vacutainer^®^, Becton Dickinson, NJ, USA). The samples were immediately centrifuged at 270× *g* for 10 min to obtain platelet-rich plasma (PRP). PRP was centrifuged at 1200× *g* for 5 min to gain platelet-poor plasma (PPP) for calibration of the aggregometer (APACT 4004 LABiTec^®^, Ahrensburg, Germany). LTA was induced using 10 µM thrombin-receptor activating peptide (TRAP)-6 as described before [31]. Results were given as the maximum of aggregation (MoA) in percent.

### 2.13. Statistical Analysis

All experiments were performed at least three times with n defined as individual animal. Data are presented as means ± SEM as indicated. Statistical analysis was performed using statistic analyzing software Graphpad Prism 8.0 (Graphpad Software, Inc., San Diego, CA, USA). Statistical tests were performed (*t*-test, one-way or two-way ANOVA) whereat a *p* < 0.05 was set as significant. For all figures * *p* < 0.05, ** *p* < 0.01, and *** *p* < 0.001.

## 3. Results

### 3.1. Enhanced Platelet Reactivity in Patients and Experimental Mice after Myocardial Infarction Causes Platelet Invasion

**Figure 1:**
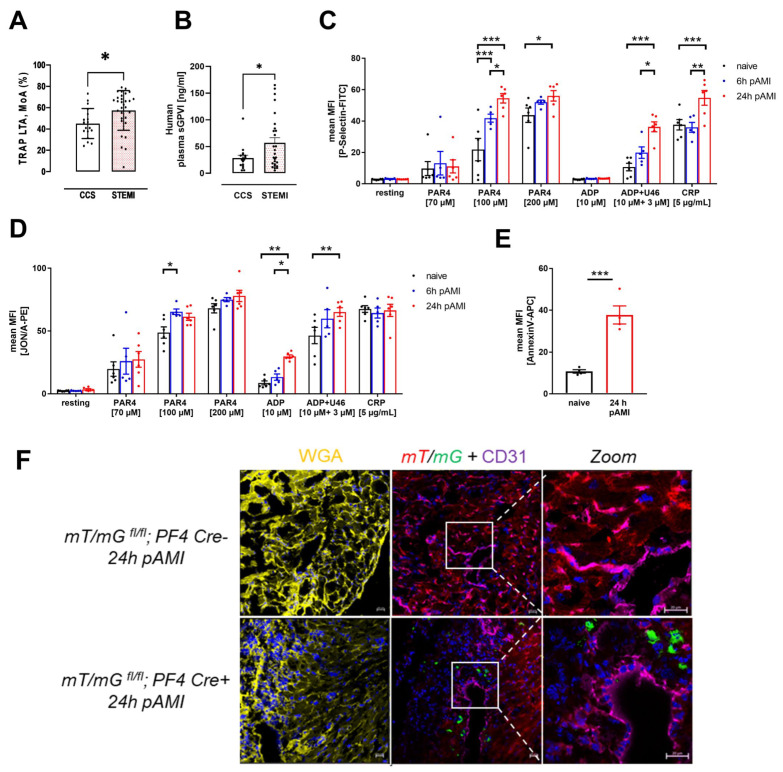
Enhanced platelet activation in STEMI patients and in a mouse model of myocardial ischemia and reperfusion. (**A**) Light transmission aggregometry was performed with TRAP stimulated platelets isolated from CCS and STEMI patients. MoA: maximum of aggregation. (**B**) Plasma analyses of soluble GPVI (sGPVI). (CCS: *n* = 17; STEMI: *n* = 16–27). (**C**) Externalization of P-selectin at the platelet surface and (**D**) activation of α_IIb_β_3_ integrin of naïve mice and 6 and 24 h post AMI at resting state or activated with indicated agonists; *n* = 6. (**E**) Annexin V binding of platelets from naive mice and 24 h post AMI; *n* = 4. (**F**) Wheat germ agglutinin (WGA) staining in yellow to identify the infarct zone by plasma membrane integrity and PECAM staining (violet) to visualize blood vessels in heart sections from mT/mG;PF4-Cre- (upper panel) and mT/mG;PF4-Cre+ mice (lower panel) 24 h post AMI. Scale bar 20 µm. Data are presented as means ± SEM. Statistical analyses were done by two-tailed unpaired Student’s *t*-test (**A**,**B**,**E**) or by Two-Way ANOVA followed by Sidak post hoc (**C**,**D**). * *p* < 0.05, ** *p* < 0.01, *** *p* < 0.001.

The analysis of platelet aggregation revealed elevated platelet activation in STEMI patients compared to patients with chronic coronary syndrome (CCS) (Figure 1A). Enhanced platelet activation was also reflected by elevated levels of soluble GPVI in the plasma of STEMI patients compared to CCS patients (Figure 1B). This prompted us to further analyze platelet activation and the consequences of elevated platelet activity after AMI in an experimental model of myocardial ischemia and reperfusion. To induce AMI in mice, the LAD was ligated for 60 min and then reperfused for 5 and 24 h to investigate the progression of ischemia and reperfusion injury. Naïve control animals obtained no surgical procedure. Platelet activation was analyzed by antibody binding to activated integrin α_IIb_β_3_ and P-selectin (marker for degranulation) on the platelet surface at indicated time points using flow cytometry (Figure 1C–D). After LAD ligation, integrin activation and P-selectin exposure was not different under non-stimulated (resting) conditions at 6 and 24 h of reperfusion. In contrast, increased P-selectin exposure was observed after stimulation of platelets with 100 µM protease-activated receptor 4 (PAR4) at both time points while only increased P-selectin was detected with 200 µM PAR4 after 24 h compared to platelets isolated from naïve mice. Furthermore, we detected enhanced P-selectin at the platelet surface when platelets were activated with ADP and U46619 or collagen related peptide (CRP) after 6 and 24 h of reperfusion (Figure 1C). Integrin activation was enhanced as well, following stimulation of platelets with 100 µM PAR4 (at 6 h of reperfusion) and with ADP or ADP and U46619 at 6 and 24 h of reperfusion, respectively (Figure 1D). Moreover, pro-coagulant activity of circulating platelets after AMI was characterized by the surface abundance of PS. As shown in Figure 1E, a significant increase in PS exposure was detected after 24 h of reperfusion (Figure 1E).

Enhanced platelet activation after 6 and 24 h of reperfusion was accompanied by migration of platelets into the infarcted myocardium. To proof the accumulation of platelets into the left ventricle after AMI, we made use of the global double fluorescent Cre reporter mouse mT/mG that has been crossbred with the megakaryocyte/platelet specific *PF4-Cre* mouse. These mice have been analyzed in detail in our lab before [32]. The mT/mG mouse expresses membrane targeted tandem dimer Tomato (mT) prior to Cre-mediated excision and membrane-targeted green fluorescent protein (mG) after excision. As a result, mT/mG; PF4-Cre+ mice exhibit green fluorescent platelets in the circulation while platelets isolated from PF4-Cre negative mice show red fluorescence [32]. To this end, mT/mG;PF4-Cre mice were analyzed under naïve conditions and after 24 h of reperfusion post cardiac ischemia. Platelets of mT/mG;PF4-Cre+ mice appeared in green in the myocardium, whereas the remaining tissue is stained in red. Immunofluorescence WGA staining (yellow) was employed to detect intact cell membranes to distinguish between the infarcted myocardium and the remote zone (Supplementary Figure S1A–C) [33]. Labeling of PECAM-1 (violet) was utilized to identify endothelial cells and to exclude those platelets that only appear in vessels but not in the cardiac tissue (Figure 1F, Supplementary Figure S1B,C). The co-staining visualized an immigration of platelets into the infarct zone of the left ventricle 24 h post AMI but not into the remote zone (Figure 1F; Supplementary Figure S1B,C). 

### 3.2. Platelet Depletion in the Early Phase after AMI Reduces Infarct Size and Improves Cardiac Function

**Figure 2:**
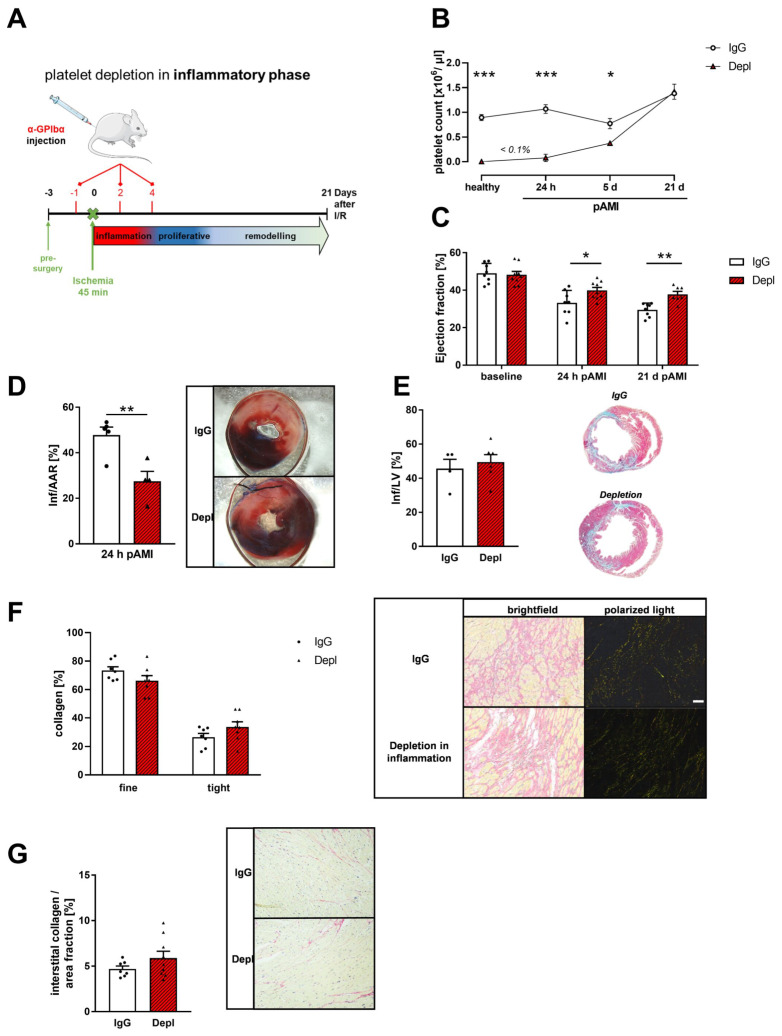
Improved cardiac function after depletion of platelets in the inflammatory phase after I/R. (**A**) Cartoon of platelet depletion and induction of I/R in mice. Mice received 2 µg/g platelet depletion antibody at indicated time points to induce platelet deletion for 48 h per injection. (**B**) Platelet counts after injection of platelet-depleted antibody in naive mice and 24 h, 5 days and 21 days post AMI compared to IgG injected control mice; *n* = 4–5. (**C**) Echocardiographic analysis of cardiac function by determination of ejection fraction (baseline vs. 24 h vs. 21 days after I/R) of IgG injected and platelet depleted mice; *n* = 8–9. (**D**) Quantitative analysis of infarct size as the percentage of area at risk (% Inf/AAR, left panel) and representative images of platelet depleted compared to IgG injected control mice 24 h post AMI (right panel). Blue = healthy tissue, red = area at risk (AAR), white = infarcted area (INF); *n* = 4–5. (**E**) Quantification (left panel) and representative images of scar size (right panel) using Gomori‘s trichrome staining 21 days post AMI. Infarcted area is stained in blue and healthy tissue is stained in red. Data are presented as means ± SEM; *n* = 4–6 (**F**) Analysis of collagen composition (left panel) and representative images of sirius-red staining (right panel) of platelet depleted mice after 21 days of reperfusion compared to their IgG injected controls, respectively; *n* = 7–8. In bright field microscopy of the LV after AMI, the cytoplasm is stained yellow and collagen is stained in red. Polarized light microscopy was used to identify thin collagen fibers (collagen type III) in green and dense collagen fibers (collagen type I) in yellow–red. (**G**) Quantification (left panel) and representative images (left panel) of sirius-red staining of interstitial collagen in the remote zone platelet depleted mice after 21 days of reperfusion compared to their IgG injected controls, respectively; *n* = 7–9. Scale bar = 50 µm. Statistical analyses were done by two-tailed unpaired Student’s *t*-test (**D**,**E**) and Two-Way ANOVA followed by Sidak post hoc (**B**,**C**,**F**,**G**). * *p* < 0.05, ** *p* < 0.01, *** *p* < 0.001.

To investigate the role and function of platelets post AMI in further detail, we depleted platelets in wildtype (WT) mice by antibody injection 24 h before LAD ligation and at day 2 and 4 post ischemia (Figure 2A,B). Control mice received an IgG antibody at the same time points before and after LAD ligation. Platelet counts were measured at different time points post AMI, demonstrating reduced platelet counts until day 5 in platelet depleted mice compared to IgG control mice (Figure 2B). In detail, naïve mice and mice that underwent ischemia and reperfusion showed >99% reduction in platelets counts 24 h post AMI (Figure 2B). After 5 days of reperfusion, the platelet count of antibody treated mice is still significantly reduced (48.6% reduction in platelet count in control mice). Platelet depletion by antibody treatment led to improved heart function indicated by increased ejection fraction at 24 h and 21 days after AMI (Figure 2C). Improved heart function was accompanied by reduced infarct size compared to IgG injected mice after 24 h of reperfusion (Figure 2D) while no alterations in scar size were detected after 21 days of reperfusion as revealed by Gomori’s trichrome staining (Figure 2E). 

The extracellular collagen composition of the infarcted myocardium is critical for the maintenance of heart function after AMI [34]. Therefore, we analyzed the collagen composition of the infarcted myocardium via picrosirius red staining and polarized light microscopy. In bright field microscopy, collagen was stained in red, and cytoplasm occurred in yellow. Due to the specific birefringence of collagen, dense fibers are stained yellow/orange in polarized light, whereas the thinner and reticular collagen fibers appear in green [35]. Platelet depletion in the early, inflammatory phase caused no significant alteration in collagen composition of the infarcted myocardium (Figure 2F). Along with unaltered collagen composition, we detected comparable amounts of interstitial collagen in the remote zone of infarcted hearts in mice (Figure 2G). 

### 3.3. Reduced Inflammatory Responses in the Acute Phase after MI in Platelet Depleted Mice

**Figure 3:**
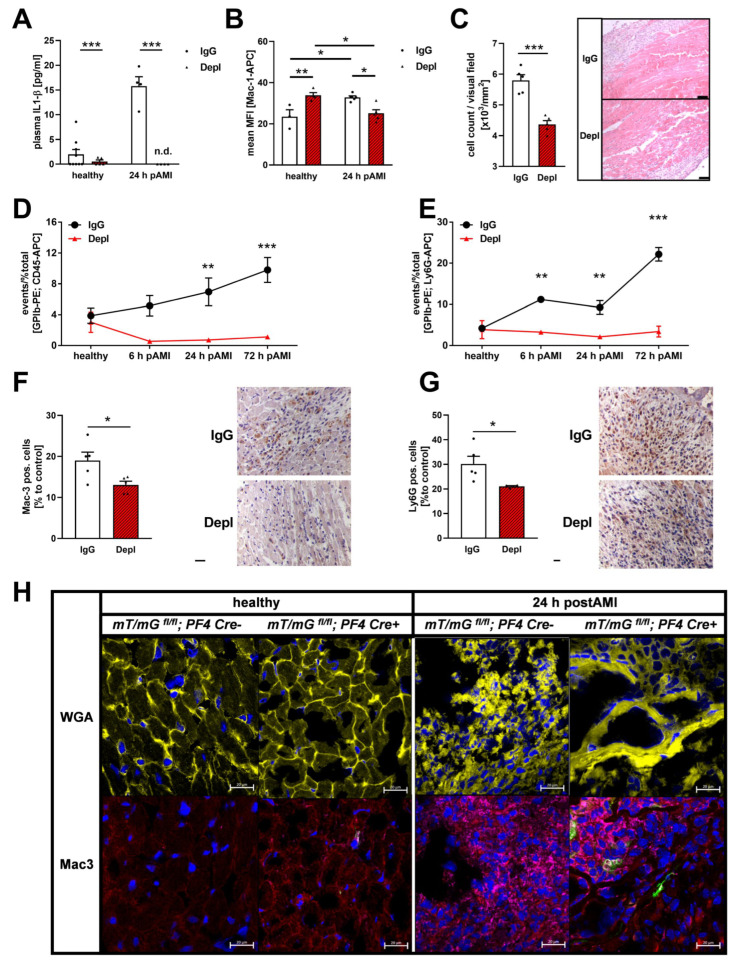
Platelet depletion leads to a reduced inflammatory response in the acute phase after myocardial infarction. (**A**) IL-1β plasma levels and (**B**) activation of neutrophils in platelet depleted naive mice and 24 h post AMI compared to IgG injected control mice. n.d. = not detectable; *n* = 4–9 (**A**); *n* = 3–6 (**B**). (**C**) Number of total cells in the infarcted myocardium in IgG injected control and platelet depleted mice 24 h post AMI; *n* = 5. (**D**,**E**) Platelet-leukocyte and platelet-neutrophils aggregates in IgG injected control and platelet depleted mice before and 6 h to 72 h post AMI; *n* = 4–6. (**F**,**G**) Number of Mac-3 (**F**) and Ly6G (**G**) positive cells in the infarcted myocardium in IgG injected control and platelet depleted mice 24 h post AMI; *n* = 5. Quantification (left panel) and representative images (right panel). (**H**) WGA staining in yellow and Mac-3 staining in violet was performed to identify macrophages in the infarcted area of the left ventricle from naïve (left panel) mT/mG;PF4-Cre- or mT/mG;PF4-Cre+ mice and 24 h post AMI (right panel), respectively. Scale bar = 50 µm. *n* = 3–4. In the utilized mouse model, platelets appear in green, whereas the remaining tissue (all other cells) shows red fluorescence. Data are presented as means ± SEM. Statistical analyses were done by Two-Way ANOVA followed by Sidak post hoc (**A**–**D**) or by two-tailed unpaired Student’s *t*-test (**E**–**G**). * *p* < 0.05, ** *p* < 0.01, *** *p* < 0.001.

The acute phase after AMI is characterized by an inflammatory response, which contributes to the healing process and scar formation. It is known that an extensive inflammatory reaction will enlarge the affected myocardial tissue after an ischemic event [36] while reduced inflammation reduced the risk of post AMI complications {Liu, 2011 #18}. To investigate the inflammatory response after AMI, platelet depleted, and IgG injected non-depleted control mice were analyzed at 24 h after ischemia and reperfusion. As expected, increased plasma levels of the acute phase cytokine IL1-β were observed 24 h post AMI in control mice. However, we were not able to detect IL1-β plasma levels in platelet depleted mice suggesting that the up-regulation of IL1-β in the plasma post AMI strictly depends on platelet activation (Figure 3A). Furthermore, enhanced Mac-1 exposition after 24 h of reperfusion indicated elevated neutrophil activation in control mice (Figure 3B). However, in platelet depleted mice, we detected enhanced Mac1 exposure on neutrophils in naïve mice but significantly reduced neutrophil activation after 24 h of ischemia reperfusion compared to IgG control (after AMI) and naïve platelet depleted mice (Figure 3B). Absent IL-1β plasma levels and reduced neutrophil activation in platelet depleted mice resulted in significantly decreased infiltration of inflammatory immune cells into the infarct border zone of platelet depleted mice (Figure 3C). Reduced platelet-leukocyte conjugates 24 and 72 h post AMI and a decreased number of GPIb-Ly6G-positive cells at 6, 24 and 72 h post AMI were detected by flow cytometry (Figure 3D,E). In line with these results, we provided evidence that reduced migration of inflammatory leukocytes includes both, macrophages and neutrophils, as indicated by less Mac-3 (Figure 3F) and Ly6G (Figure 3G) positive cells in the infarcted myocardium after 24 h of ischemia and reperfusion as revealed by histological analysis. 

In mT/mG;PF4-Cre+ mice, an MI-induced increment of macrophages was identified by Mac3 (violet) staining in the myocardium after 24 h of reperfusion while no macrophages were detected in the heart of naïve mice (Figure 3H). Interestingly, platelets (green) accumulate around the migrated macrophages (violet) in the infarct zone 24 h after AMI (Figure 3H).

### 3.4. Platelets Modulate Cardiac Remodeling and Scar Formation after AMI

**Figure 4:**
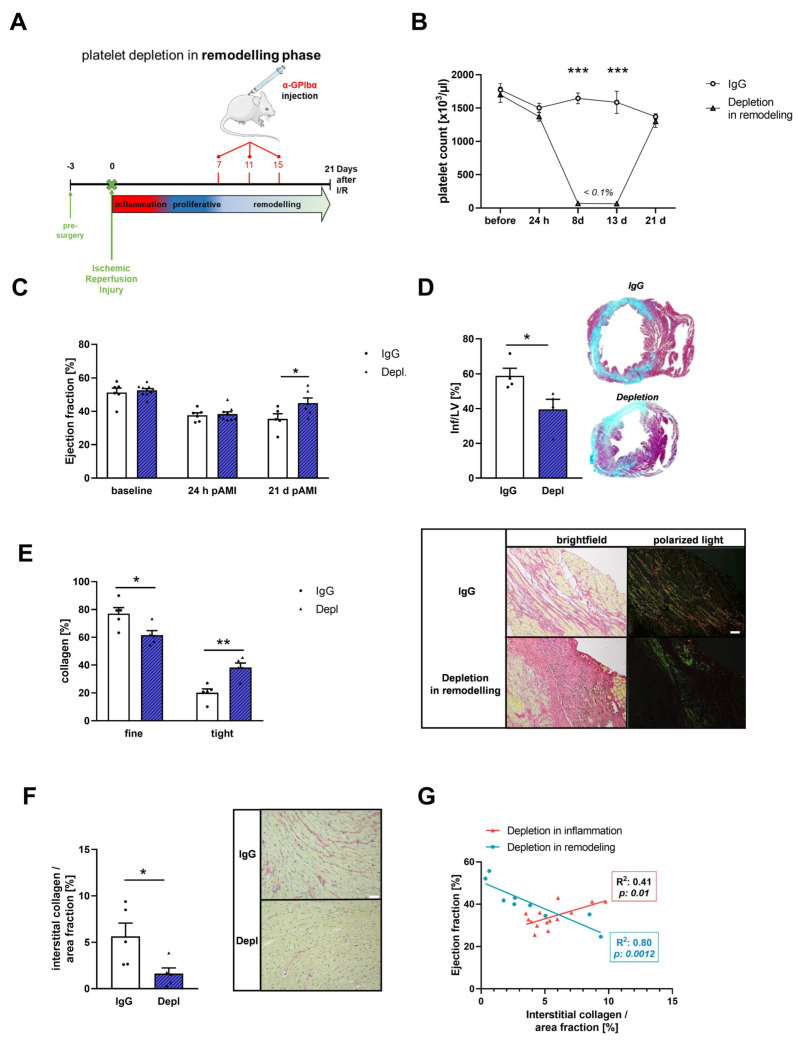
Platelet depletion in the remodeling phase after ischemic/reperfusion injury results in altered scar formation. (**A**) Time schedule of platelet depletion and induction of I/R in mice. Platelets were depleted in the remodeling phase and compared to IgG injected controls. Mice received 4 µg/g platelet depletion antibody at indicated time points to induce platelet deletion for 96 h per injection. (**B**) Platelet counts at different time points. *n* = 5–8 (**C**) Echocardiographic analysis of ejection fraction (baseline vs. 24 h vs. 21 days after I/R) of IgG injected and platelet depleted mice; *n* = 5–10. (**D**) Quantification (left panel) and representative images of scar size (right panel) using Gomori‘s trichrome staining 21 days post AMI. *n* = 3–4. Infarcted area is stained in blue and healthy tissue is stained in red. Data are presented as means ± SEM. (**E**) Analysis of collagen composition (left panel) and representative images of sirius-red staining (right panel) of platelet depleted mice after 21 days of reperfusion compared to their IgG injected controls, respectively; *n* = 5. (**F**) Quantification (left panel) and representative images (left panel) of sirius-red staining of interstitial collagen in the remote zone platelet depleted mice after 21 days of reperfusion compared to their IgG injected controls, respectively; *n* = 5. Scale bar = 50 µm. (**G**) Spearman correlation was determined, as indicated. Spearman correlation between ejection fraction and interstitial collagen content was determined. Rho (r) = −1 indicates strong negative correlation, r = 0 indicates no correlation and r = +1 indicates strong positive correlation. *p*-values, as indicated. Data are presented as means ± SEM. Statistical analyses were done by two-tailed unpaired Student’s *t*-test (**D**,**F**) and Two-Way ANOVA followed by Sidak post hoc (**B**,**C**,**E**). * *p* < 0.05, ** *p* < 0.01, *** *p* < 0.001.

For a detailed analysis of platelets and their role in cardiac remodeling, we depleted platelets in the remodeling phase (platelet depletion between day 7 and 15 post I/R) after AMI (Figure 4A,B). Scar size, collagen composition and interstitial collagen content were investigated by histology at 21 days of reperfusion, respectively. First, Gomori’s trichrome staining revealed that loss of platelets in the remodeling phase improved cardiac function as reflected by enhanced ejection fraction (Figure 4C) and led to significantly reduced scar size compared to the non-depleted controls (Figure 4D). Next, we analyzed the collagen composition of the infarcted myocardium via picrosirius red staining and polarized light microscopy. In contrast to platelet depletion in the early, inflammatory phase (Figure 2F), we detected significant alterations in collagen composition of the infarcted myocardium when we depleted platelets in the remodeling phase. In detail, we found less thin collagen and denser collagen in the infarcted area of the LV after 21 days of reperfusion (Figure 4E). Along with changes in collagen composition, we detected significantly reduced interstitial collagen in the remote zone of infarcted hearts in mice, where platelet depletion was initiated in the remodeling phase (Figure 4F). These different results in interstitial collagen content between platelet depletion in the early, inflammatory and in the remodeling phase, were reflected by Spearman correlation analysis that identifies a positive correlation between interstitial collagen content and improved ejection fraction when platelets were depleted in the remodeling phase (Figure 4G). 

### 3.5. Platelet Activation Affects Cardiac Fibroblast Transformation in the Remodeling Phase after AMI

**Figure 5:**
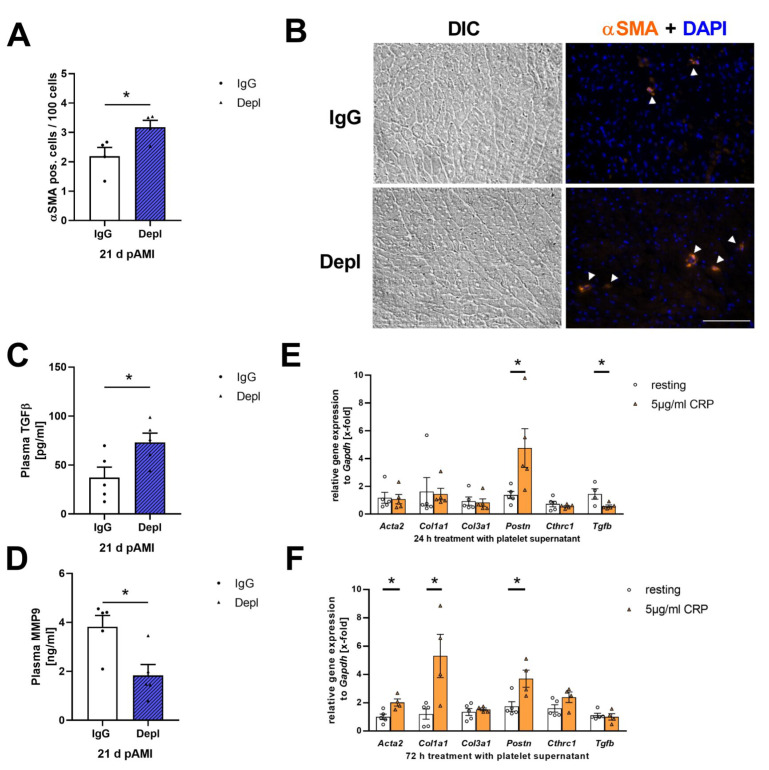
Platelets activation affect cardiac fibroblast activation and transformation in the remodeling phase after AMI. (**A**) Quantification and (**B**) representative images of αSMA positive cells (white arrows) in the infarcted myocardium of platelet depleted mice after 21 days of reperfusion compared to IgG injected control mice; *n* = 4. (**C**,**D**) Plasma levels of (C) TGF-β and (**D**) MMP9 after 21 days of reperfusion. *n* = 5. (**E**,**F**) Gene expression of *Acta2*, *Col1a1*, *Col3a1*, *Postn*, *Cthrc1* and *Tgfβ* in isolated cardiac fibroblasts from wildtype mice treated with the supernatant of resting and CRP (5 µg/mL) activated platelets for (**E**) 24 h or (**F**) 72 h; *n* = 4–5. Scale bar = 100 µm. Data are presented as means ± SEM. Statistical analyses were done by two-tailed unpaired Student’s *t*-test. * *p* < 0.05.

Collagen is mainly synthesized by activated fibroblasts [37]. MI induces fibroblast activation in the infarcted myocardium [38]. After an ischemic event, resting fibroblasts become activated and transdifferentiate to myofibroblasts that are characterized by a contractile phenotype with enhanced expression of α-smooth muscle actin (αSMA) and elevated secretion of ECM proteins [39]. Due to the observed changes in collagen composition, we investigated the number of αSMA positive cells in heart sections of mice where platelets have been depleted in the remodeling phase. Platelet depletion led to an increase in αSMA positive cells within the infarcted myocardium compared to IgG injected control mice (Figure 5A,B). Transforming growth factor-β (TGF-β) is the most common platelet-derived stimulator of αSMA expression and fibroblast activation [40,41]. Platelet depletion in the remodeling phase showed a significant elevation of TGF-β in plasma 21 days post I/R (Figure 5C). In contrast, TGF-β plasma levels in the acute phase after AMI were significantly reduced in platelet depleted mice (Supplementary Figure S2). However, plasma levels of matrix metalloproteinase-9 (MMP-9), an ECM degrading enzyme, were decreased 21 days post I/R (Figure 5D).

Since we detected platelets in the infarct border zone after AMI, suggesting active migration of platelets after ischemia, we investigated the influence of platelets on fibroblast activation in further detail. To this end, we isolated primary cardiac fibroblasts and stimulated these cells with the supernatant of resting and activated platelets. After 24 h of incubation, gene expression of periostin (*Postn*), an important matricellular factor, was upregulated after incubation of cardiac fibroblasts with the supernatant of CRP activated platelets (Figure 5E). In contrast, *Tgfb1* expression was decreased after stimulation with activated platelet supernatant, suggesting that platelets inhibit the expression and release of TGF-β of fibroblasts (Figure 5E). However, after 72 h the gene expression of *Acta2*, *Col1a1* and *Postn* were enhanced in primary fibroblasts stimulated with activated platelet supernatant, indicating enhanced cardiac fibroblast activation induced by activated platelet supernatant (Figure 5F). 

### 3.6. Reduced Platelet-Mediated Inflammation in Mice with Chronic Thrombocytopenia

**Figure 6:**
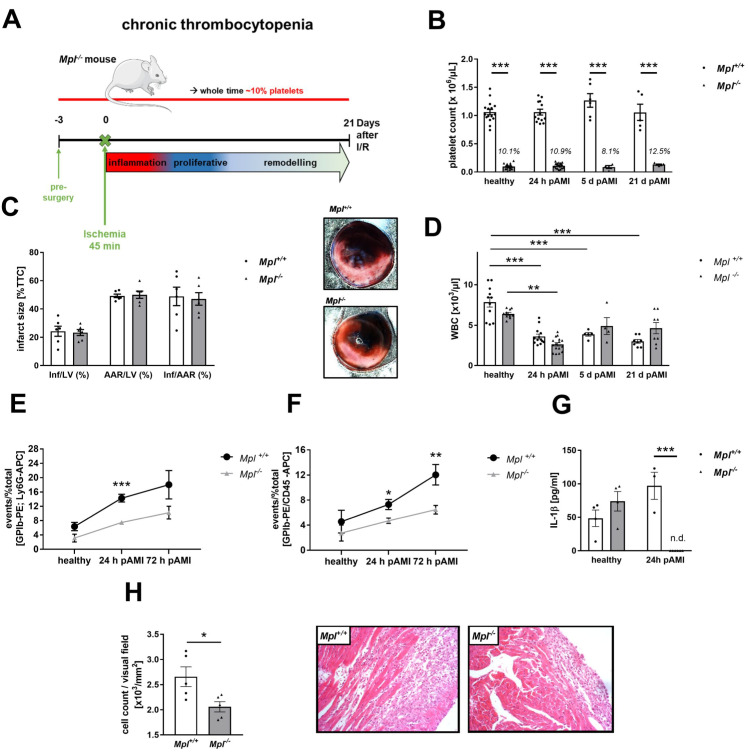
Mice with chronic thrombocytopenia show absent IL-1β plasma levels and reduced migration of inflammatory cells into the infarct border zone. (**A**) Schematic illustration of chronic thrombocytopenia in genetically modified mice (*Mpl^−/−^*). (**B**) Platelet counts in naïve MPL deficient mice and at indicated time points after I/R. *n* = 16 (naïve mice), *n* = 14–16 (24 h post AMI), *n* = 4–6 (5d post AMI), *n* = 5 (21d post AMI). (**C**) Infarct size was not altered in MPL deficient mice 24 h post AMI. Quantification of infarct size (left panel) and representative images (right panel). (**D**) White blood cell counts at indicated time points in chronic thrombocytopenic mice. (**E**,**F**) platelet-neutrophils and platelet-leukocyte conjugates in MPL deficient and control mice before and 24 h to 72 h post AMI; *n* = 5–6. (**G**) IL-1β plasma levels in naive mice and 24 h and 21d post AMI; *n* = 3–4. (**H**) Number of inflammatory cells in the infarcted myocardium in MPL deficient and control mice 24 h post AMI; *n* = 5. Quantification (left panel) and representative images (right panel). Data are presented as means ± SEM. Statistical analyses were done by Two-Way ANOVA followed by Sidak post hoc. * *p* < 0.05, ** *p* < 0.01, *** *p* < 0.001.

To further analyze the impact of platelets in the inflammatory response post AMI, we used a mouse model with genetically induced thrombocytopenia, the *Mpl^−/−^* mice. These mice display platelet counts of 8.1–12.5% compared to control mice (Figure 6A,B). After 24 h of reperfusion, infarct size, as detected by TTC staining, was unaltered between MPL deficient and control mice (Figure 6C). Unaltered white blood cell (WBC) counts between *Mpl^−/−^* and control mice were detected in healthy individuals. However, WBC counts drop significantly in both groups 24 h post AMI, probably due to invasion of inflammatory leukocytes into the infarct border zone (Figure 6D). In contrast to platelet depleted mice, where platelet-leukocyte conjugates were almost absent, we were able to detect moderate platelet-leukocyte and platelet-neutrophil conjugates in *Mpl^−/−^* and control mice after 24 and 72 h of reperfusion (Figure 6E,F). However, *Mpl^−/−^* mice showed significantly less conjugates after AMI compared with control mice. In line with platelet depleted mice, we found almost absent IL-1β plasma levels in *Mpl^−/−^* mice (Figure 6G) and significantly reduced migration of leukocytes into the infarct border zone 24 h post AMI compared to control mice (Figure 6H). 

### 3.7. Unaltered Collagen Composition and Left Ventricular Function in Mice with Chronic Thrombocytopenia

**Figure 7:**
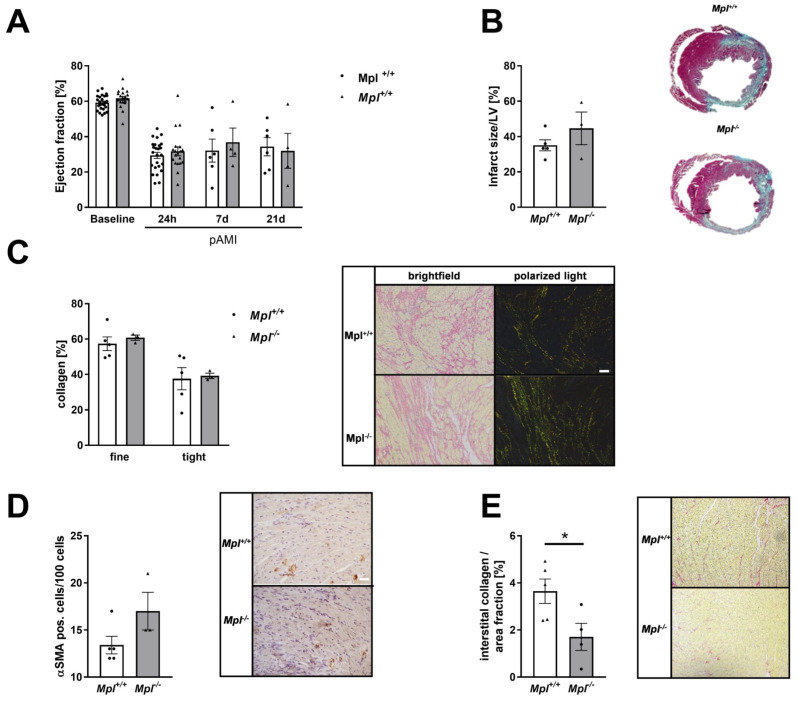
Unaltered collagen composition and LV function in MPL deficient mice after I/R. (**A**) Echocardiographic analysis of cardiac function by determination of ejection fraction (baseline vs. 24 h, 7d and 21 d after I/R) of MPL deficient and control mice; *n* = 19–25 (basline and 24 h post AMI), *n* = 5 (7d and 21d post AMI). (**B**) Quantification (left panel) and representative images of scar size (right panel) using gomori‘s trichrome staining 21 days post AMI. *n* = 3–4. Infarcted area is stained in blue and healthy tissue is stained in red. Data are presented as means ± SEM. (**C**) Analysis of collagen composition (left panel) and representative images of sirius-red staining (right panel) of MPL deficient and control mice after 21 days of reperfusion; *n* = 3–4. (**D**) Quantification of αSMA positive cells in the infarcted myocardium of MPL deficient mice after 21 days of reperfusion compared to control mice; Quantification (left panel) and representative images (right panel). *n* = 4. (**E**) Quantification (left panel) and representative images (left panel) of sirius-red staining of interstitial collagen in the remote zone of MPL deficient and control mice after 21 days of reperfusion; *n* = 5–9. Scale bar = 50 µm. Statistical analyses were done by Two-Way ANOVA followed by Sidak post hoc. * *p* < 0.05.

In genetically induced thrombocytopenic mice, we detected no alterations in left ventricular function and scar size in *Mpl^−/−^* mice compared to controls (Figure 7A,B). In contrast to platelet depleted mice, *Mpl^−/−^* mice showed no differences in collagen content or αSMA positive cells in the infarcted myocardium (Figure 7C,D). However, we detected significantly reduced interstitial collagen in the remote zone of infarcted hearts in *Mpl^−/−^* mice (Figure 7E).

## 4. Discussion

In this study, we found that AMI induces elevated platelet activation in mice and patients, leading to platelet invasion into the infarct zone. Platelets are able to modulate inflammation and cardiac remodeling by interaction with inflammatory cells and cardiac fibroblast. The absence of platelets, either due to acute or chronic thrombocytopenia, led to a reduced inflammatory response in the acute state after MI. However, only acute thrombocytopenia alters cardiac remodeling that, together with reduced inflammation, led to decreased infarct/scar size and improved left ventricular function. In contrast, chronic thrombocytopenia in MPL deficient mice did not change cardiac remodeling, leading to scar formation and cardiac damage comparable to control mice. Thus, left ventricular function was not altered in MPL deficient mice. 

Platelet activation during AMI is associated with coronary occlusion and myocardial injury. It has been shown that P-selectin expression by activated platelets contributes to I/R injury [15]. Therefore, the use of antiplatelet agents, such as aspirin and ADP receptor antagonists, remain essential for the prevention of ACS [42]. Different studies in the past provided evidence for P-selectin to be a critical mediator [15,43,44,45]. Here, we show that platelets isolated from mice with AMI have an enhanced activation profile following stimulation of platelets with different agonists (Figure 1). Platelet activation is enhanced following stimulation with different agonists at early (6 h, 24 h) time points. Likewise, platelets from STEMI patients showed enhanced platelet aggregation in response to the Protease-activated receptor (PAR)1 agonist TRAP due to enhanced platelet activation. Since mouse platelets do not express PAR1, we activated the thrombin receptor PAR4 and could show that also PAR4 is involved in platelet activation after I/R because enhanced integrin activation and P-selectin exposure was observed after 4 and 24 h post I/R. Thus, it is not surprising that PAR4 deficiency plays a crucial role in cardioprotection after AMI, at least in mice [16]. Cardioprotection was mediated in part by reduced inflammation in PAR4 deficient mice following AMI [16]. 

P-selectin was identified to play an important role in the inflammatory response after I/R, as well. The number of P-selectin positive platelets was enhanced 24 and 72 h post I/R in mice important to mediated platelet-leukocyte conjugates after AMI. An antibody against PSGL1 or treatment with Clopidogrel reduced the number of P-selectin positive platelets and platelet-leukocyte conjugates, emphasizing the role of P-selectin in platelet activation and inflammation. The same group also showed reduced platelet-leukocyte conjugates and reduced inflammatory cell recruitment into the infarcted myocardium when they depleted platelets in mice or after treatment with antiplatelet therapy. However, they do not analyze the effects of reduced acute inflammation on infarct size or LV function. Here, we show that acute thrombocytopenia by antibody-induced platelet depletion improved heart function at early and even at late time points after I/R that might be due -at least in part- to reduced inflammation (Figure 2). However, cardiac remodeling and scar size was only improved/reduced upon acute thrombocytopenia because platelet depletion in the inflammatory phase and chronic thrombocytopenia in MPL deficient mice did not affect collagen composition. This strengthens the hypothesis that only acute thrombocytopenia is beneficial for improved scar formation and left ventricular function. However, our data confirms that platelet mediated inflammation plays a crucial role for the extent of cardiac damage and repair and is a key event in atherothrombosis providing a link between thrombosis and inflammation (thrombo-inflammation) [46] because platelet depletion in the inflammatory phase did not only improve left ventricular function after 24 h but also after 21 days (Figure 2). Interestingly, the number of circulating monocyte-platelet aggregates has been shown to be a more sensitive marker of in vivo platelet activation than the exposure of P-selectin at the platelet surface [47]. 

Results from different groups provide strong evidence that the extracellular collagen matrix plays an important role in the healing and remodeling process after AMI [48]. Here, we could demonstrate a pivotal role of platelets in this process because platelets are able to activate primary cardiac fibroblasts by the release of active substances in vitro (Figure 5) and acute thrombocytopenia alters scar formation leading to reduced scar size and improved heart function 21 d post AMI (Figure 4). Thus, our data clearly indicates a dominant role for platelets in the remodeling process after I/R. Platelets modulate gene expression of cardiac fibroblasts, leading to reduced *Tgf-β* and enhanced *Acta2* and *ColI* expression in vitro. In line with these results, Yabanoglu and colleagues found an upregulation of *Acta2* in rat cardiac fibroblasts when they were treated with platelet lysates [49]. This effect was accompanied by enhanced migration and proliferation of cardiac fibroblasts and elevated TGF-β levels that might be—at least in part–of platelet origin. In contrast, our data showed enhanced TGF-β plasma levels in platelet depleted mice 21 d post AMI. This suggests that TGF-β in platelet depleted mice might be of fibroblast origin because the incubation of cardiac fibroblasts with platelet releasates led to reduced TGF-β gene expression. In contrast, reduced TGF-β plasma levels were detected in platelet depleted mice 5 d post AMI that might be of platelet origin. Improved scar formation in platelet depleted mice might be due to enhanced TGF-β plasma levels, an enhanced number of αSMA positive fibroblasts and reduced MMP9 levels together with reduced collagen I expression of fibroblasts. The importance of αSMA positive myofibroblasts for scar contraction was already shown before [50]. Furthermore, the reduction in collagen I but not collagen III expression of fibroblasts in thrombocytopenic mice might change the LV collagen type I/III ratio after AMI leading to enhanced collagen III that might be responsible for enhanced dense collagen and reduced thin collagen 21 d post AMI. These effects are in line with results from different groups demonstrating an improved cardiac function by collagen III overexpression and a correlation of collagen I levels, with LV dysfunction [51,52]. A study from Xie and colleagues further supports the importance of the collagen type I/III ratio for scar formation because an increase in the ratio could make the deposited collagens stiffer and enhance the cross-linking capacity and decreased the degradation process [53]. Furthermore, enhanced TGF-β plasma levels in platelet depleted mice might protect cardiomyocytes from ischemic damage because Walsh and Poole already found that the α-granule components SDF-1α and TGF-β exert protective effects on cardiomyocytes in vitro. Importantly, they provide evidence for P2Y_12_ antagonists to attenuate the protective effects of platelet components [20]. 

The results from the two different thrombocytopenic mouse models used in this study emphasizes that only acute thrombocytopenia is protective in AMI. Different causes have been described to induce thrombocytopenia: Primary and drug-induced immune thrombocytopenia, infections such as HIV, sepsis, etc., chronic alcohol abuse, nutrient deficiencies, autoimmune disorders, pregnancy, cancer, surgery and inherited thrombocytopenia such as von Willebrand factor disease, Bernard-Soulier syndrome etc. [54]. Thrombocytopenia can be associated with bleeding risks and thrombosis (in conditions like heparin-induced thrombocytopenia, HIT; disseminated intravascular coagulation (DIC), etc.). To date, the results about the outcome of AMI in thrombocytopenic patients are diverse and only a limited number of studies exist. In immune thrombocytopenia purpura (ITP), two distinct clinical syndromes manifest as an acute condition in children and a chronic condition in adults. ITP patients with AMI have an increased risk of bleeding and more cardiovascular complications with a similar in-hospital mortality risk [55]. Another study from Davis and colleagues provided evidence that low platelet counts may exert protective effects from STEMI because ITP patients developed lower STEMI rates [12]. In contrast, Rubinfeld and colleagues found elevated bleeding complications, cardiovascular outcomes and mortality in patients with thrombocytopenia, suggesting a poor prognosis for these patients after AMI [11]. We hypothesize that differences in cardiac remodeling, scar formation and left ventricular function might be due to acute versus chronic thrombocytopenia. However, the total platelet counts between (thrombocytopenic) patients might be also a relevant marker since differences in platelet counts between platelet depleted mice (platelet count <1% compared of controls) and MPL deficient mice (platelet count ~10%) might account for differences in cardiac remodeling and left ventricular function. This is strengthened by a patients study from Chao and colleagues who believe that thrombocytopenia should be considered as a surrogate marker of poor prognosis in patients with acute coronary syndrome because all-cause mortality increased with the severity of thrombocytopenia [56]. Thus, our study comparing the outcome of AMI under acute and chronic thrombocytopenia in mice provides a first hind for the different results published by different groups. Besides, different results from our group published in the last years indicate that alterations in the inflammatory response alone are not sufficient to effect infarct size or left ventricular function after AMI [57,58]. However, further investigations are necessary to analyze the impact of thrombocytopenia on AMI and to improve the prognosis of patients with AMI and thrombocytopenia.

Taken together, we here show that platelets play a crucial role in inflammation and cardiac remodeling post AMI. Acute and chronic thrombocytopenia exert different outcomes in mice with AMI, with only a cardio-protective effect in mice with acute thrombocytopenia. However, the type of thrombocytopenia and total platelet counts might be relevant for left ventricular function and the prognosis of patients with AMI. Thus, further studies are needed to optimize antithrombotic/antiplatelet strategies in AMI.

## Data Availability

Data are available from the corresponding author upon request.

## Supplementary Materials

The following supporting information can be downloaded at: https://www.mdpi.com/article/10.3390/cells11213500/s1, Figure S1: Platelet accumulation in the infarct zone 24 h post I/R. Cryo heart sections of mT/mG;PF4-Cre- or mT/mG;PF4-Cre+ mice were analyzed for the presence of platelets. (A,B) Wheat germ agglutinin (WGA) staining in yellow allows to distinguish between the infarct and the remote zone by plasma membrane integrity at 24 h after I/R. (B) PECAM staining (violet) was utilized to visualize blood vessels in heart sections from mT/mG;PF4-Cre- or mT/mG;PF4-Cre+ mice at 24 h post AMI. Green, fluorescent platelets were detected in the infarct zone within cardiac tissue (green = platelets; red = cardiomyocytes; DAPI = nucleus; violet = PECAM). (C) Wheat germ agglutinin (WGA) staining in yellow to identify the infarct zone by plasma membrane integrity and PECAM staining (violet) to visualize blood vessels in heart sections from healthy (left panel) mT/mG;PF4-Cre- or mT/mG;PF4-Cre+ mice or after 24 h post AMI (right panel). Scale bar 20 μm. (D) IgG control experiments were performed to confirm the specificity of the PECAM antibody; Figure S2: TGF-β plasma levels in platelet depleted mice at early time points after AMI. (A) Plasma levels of TGF-β from IgG injected control and platelet depleted mice were determined by ELISA using plasma from naive mice and mice that underwent AMI. Statistical analyses were done by two-tailed unpaired Student’s *t*-test (*n* = 5), ** *p* < 0.01, *** *p* < 0.001.
