# Supplementary material for: Only Acute but Not Chronic Thrombocytopenia Protects Mice against Left Ventricular Dysfunction after Acute Myocardial Infarction
Source: Cells. 2022 Nov 4;11(21):3500. doi: 10.3390/cells11213500 (PMC9659072; doi:10.3390/cells11213500)
Supplement: Supplementary file 1 [file cells-11-03500-s001.zip › cells-1906368-supplementary.pdf]

SUPPLEMENTAL FIGURES

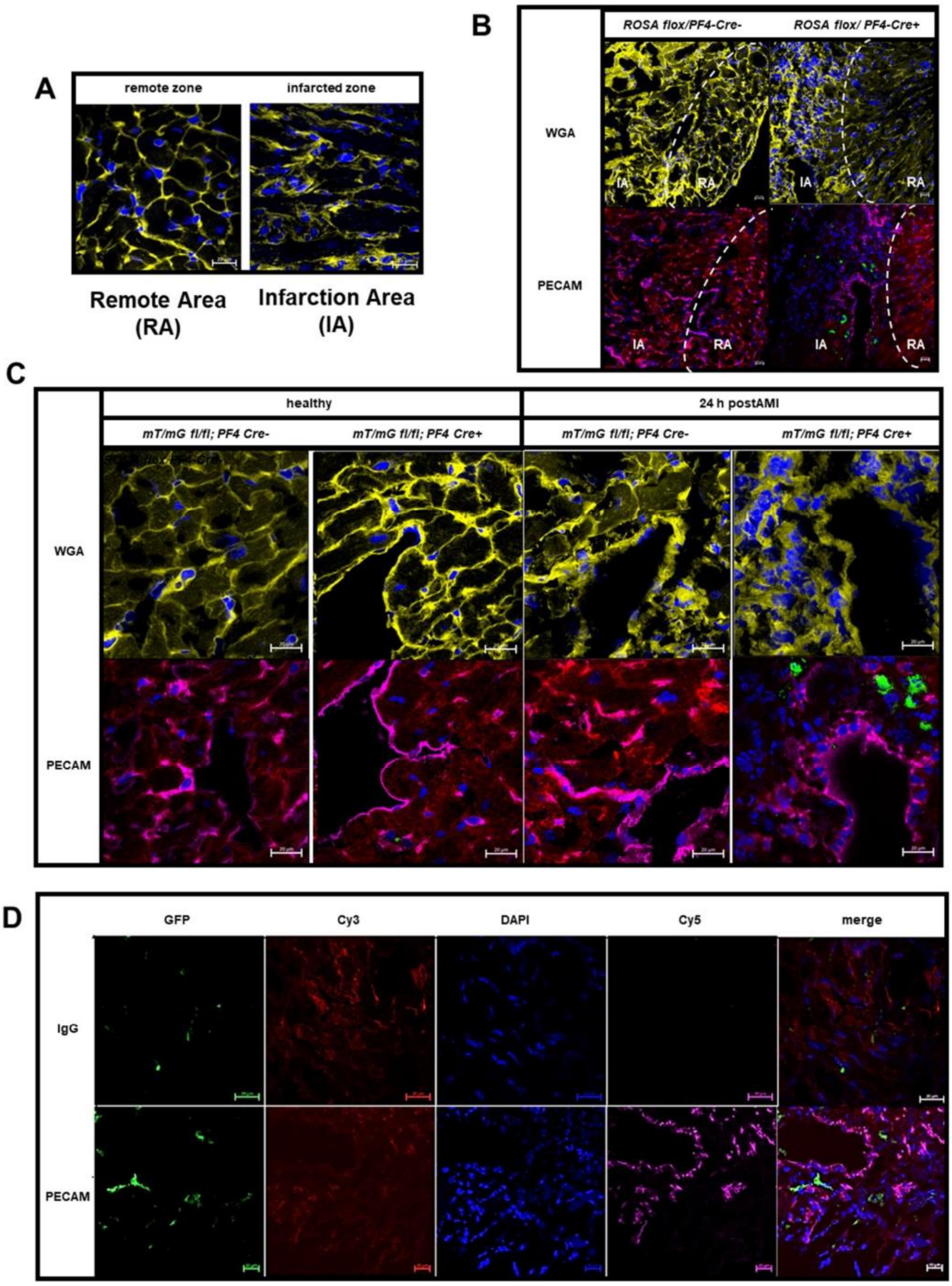

Supplementary Figure S1: Platelet accumulation in the infarct zone 24h post I/R. Cryo heart sections of mT/mG;PF4-Cre- or mT/mG;PF4-Cre+ mice were analyzed for the presence of platelets. (A-B) Wheat germ agglutinin (WGA) staining in yellow allows to distinguish between the infarct and the remote zone by plasma membrane integrity at 24h after I/R. (B) PECAM staining (violet) was utilized to visualize blood vessels in heart sections from mT/mG;PF4-Cre- or mT/mG;PF4-Cre+ mice at 24 h post AMI. Green fluorescent platelets were detected in the infarct zone within cardiac tissue (green = platelets; red = cardiomyocytes; DAPI = nucleus; violet = PECAM). (C) Wheat germ agglutinin (WGA) staining in yellow to identify the infarct zone by plasma membrane integrity and PECAM staining

(violet) to visualize blood vessels in heart sections from healthy (left panel) mT/mG;PF4-Cre- or mT/mG;PF4-Cre+ mice or after 24 h post AMI (right panel). Scale bar 20 µm. (D) IgG control experiments were performed to confirm the specificity of the PECAM antibody.

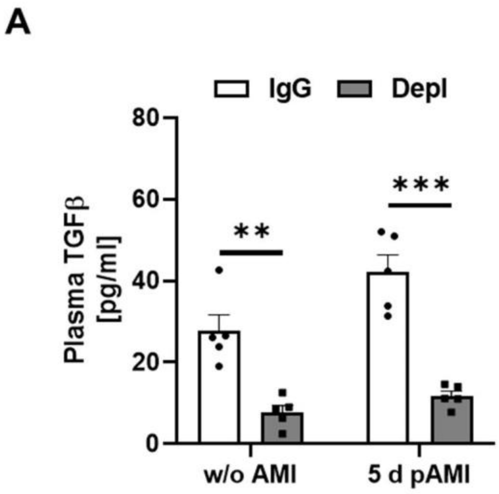

Supplementary Figure S2: TGFE plasma levels in platelet depleted mice at early time points after AMI. (A) Plasma levels of TGFE from IgG injected control and platelet depleted mice were determined by ELISA using plasma from naive mice and mice that underwent AMI. Statistical analyses were done by two-tailed unpaired Student's t-test (n = 5), **$p < 0.01$, ***$p < 0.001$.
